# Freestanding region-responsive bilayer for functional packaging of ingestible devices

**DOI:** 10.1038/s41378-023-00536-w

**Published:** 2023-05-16

**Authors:** Michael A. Straker, Joshua A. Levy, Justin M. Stine, Vivian Borbash, Luke A. Beardslee, Reza Ghodssi

**Affiliations:** 1 Fischell Department of Bioengineering, University of Maryland, College Park, MD 20742 USA; 2 Institute for Systems Research, University of Maryland, College Park, MD 20740 USA; 3 Robert E. Fischell Institute for Biomedical Devices, University of Maryland, College Park, MD 20850 USA; 4 Department of Material Science and Engineering, University of Maryland, College Park, MD 20740 USA; 5 Department of Electrical and Computer Engineering, University of Maryland, College Park, MD 20742 USA

**Keywords:** Nanoscience and technology, Nanoscale materials, Engineering

## Abstract

Ingestible capsules have the potential to become an attractive alternative to traditional means of treating and detecting gastrointestinal (GI) disease. As device complexity increases, so too does the demand for more effective capsule packaging technologies to elegantly target specific GI locations. While pH-responsive coatings have been traditionally used for the passive targeting of specific GI regions, their application is limited due to the geometric restrictions imposed by standard coating methods. Dip, pan, and spray coating methods only enable the protection of microscale unsupported openings against the harsh GI environment. However, some emerging technologies have millimeter-scale components for performing functions such as sensing and drug delivery. To this end, we present the freestanding region-responsive bilayer (FRRB), a packaging technology for ingestible capsules that can be readily applied for various functional ingestible capsule components. The bilayer is composed of rigid polyethylene glycol (PEG) under a flexible pH-responsive Eudragit^®^ FL 30 D 55, which protects the contents of the capsule until it arrives in the targeted intestinal environment. The FRRB can be fabricated in a multitude of shapes that facilitate various functional packaging mechanisms, some of which are demonstrated here. In this paper, we characterize and validate the use of this technology in a simulated intestinal environment, confirming that the FRRB can be tuned for small intestinal release. We also show a case example where the FRRB is used to protect and expose a thermomechanical actuator for targeted drug delivery.

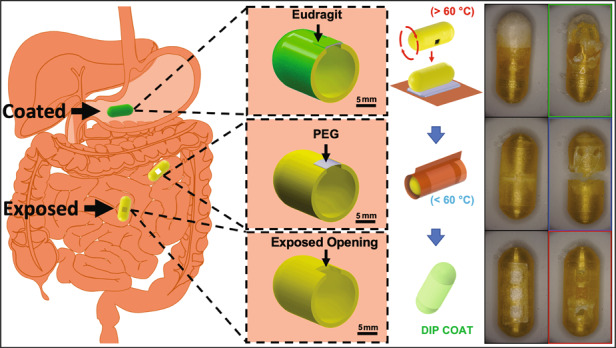

## Introduction

Ingestible devices are becoming an attractive solution for overcoming the limitations of traditional means of diagnosis and intervention for many health conditions, including inflammatory bowel disease (IBD), which affects 6.8 million people worldwide^1–3^. Currently available ingestible capsules, such as Pillcam^1,4,5^, SmartPill^6–8^, and IntelliCap^9–11^, are capable of general regional drug release and pH, temperature, pressure, and optical sensing in regions of the gastrointestinal tract (GIT) that endoscopes are not, such as in the small intestine. The next generation of ingestible devices will be imbued with functional components that interface with the target environment to perform complex diagnostic and therapeutic functions. However, to perform these tasks, the components of ingestible devices must be protected from gastrointestinal (GI) fluids or contents until they arrive at the intended site of action.

A common solution to this issue is the use of pH-responsive surface coatings to passively mediate region-specific targeting^12–15^. Commercially available pH-responsive polymer coatings have been utilized in the packaging of ingestible capsules to facilitate selective access of drug delivery^13^, sensing^14,15^, and sample collection components to the GI environment^16,17^. These coatings are commonly used in the pharmaceutical industry for the targeted release of drugs in specific GI regions^18,19^. Additionally, they have been widely adapted for use with multiscale therapeutic carriers for pH-specific targeting^20–28^. This methodology leverages the expansion and contraction of pH-sensitive materials when ionized in response to changes in ambient pH. Enteric coatings utilize polyacids, i.e., polymers with functional groups that ionize above a pH threshold, to enable regional targeting. For instance, in the acidic gastric environment (pH 1.5–3), these coatings remain in a contracted state, preventing fluid transport across the coating^29–31^. Upon arrival in the small intestine (pH 6.4–7.5) or large intestine (pH 6.4–7), the polymer expands and delivers the payload via dissolution- or diffusion-mediated mechanisms^9,32^. Enteric coatings have been applied to ingestible capsules to passively interface with the GI environment for drug delivery and sampling applications, including dissolution for passive region-specific actuator release^13,33^ and the collection of ambient media for sample analysis^12^. However, these approaches do not lend themselves to millimeter-scale actively triggered or site-specific systems, which would require the structure or microscale mesh-like openings of the supporting materials to be maintained to avoid component damage during the standard coating process.

Our group previously demonstrated an ingestible device utilizing a standard commercially available enteric coating (Eudragit® L100) for the detection of GI disease biomarkers in the small intestine^14,15^. The coating of these ingestible capsules was achieved through dip coating, although other coating methods, such as spray coating^34,35^ and pan coating^36,37^, are also frequently utilized in pharmaceutical applications. While these alternative techniques may have higher throughput and produce more precise, uniform coating layers, they also require specialized equipment and may not be suitable for electronic capsule coating. Additionally, to dip coat capsules containing functional component ports on the millimeter scale, ultrahigh-viscosity coating solutions are needed, resulting in excessively thick coating layers and impractically slow removal and release times when compared with intestinal transit^14,38–44^. Currently, the only alternative to enteric coatings for selectively exposing capsule components, such as sensors and actuators, is the addition of an active mechanical opening mechanism. Such mechanisms may require external stimuli from high-powered external equipment or the consumption of precious capsule space and energy, which are limited due to size and power source capacity^45^.

**Fig. 1:**
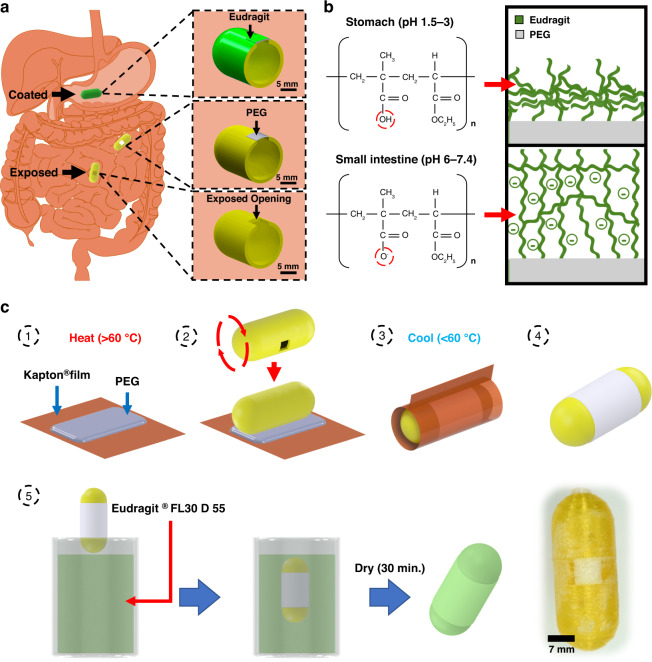
Overview of the application of the packaging system. **a** pH-sensitive polymer coating (green) remains intact and impermeable in the stomach (pH 1.5–3). In the small intestine, the pH rises (pH 6–7.4), causing polymer swelling and removal. The water-soluble PEG layer is then dissolved by the aqueous intestinal environment, thus revealing the capsule actuator cavity. **b** Chemical structure of Eudragit L 100 55 at pH values relevant to the stomach and small intestine. In the stomach, the structure is protonated, and the polymer is compact, while in the small intestine, the structure is deprotonated, causing monomers to repel each other and expand. **c** Overview of the bilayer coating process: (1) A polyimide sheet is placed on a hotplate, and PEG crystals are melted into a liquid film. (2) A 3D-printed polyethylene terephthalate glycol capsule is pressed onto a PEG film. (3) The polyimide and PEG layers are folded over the capsule and cooled until the PEG film solidifies. (4) The polyimide film layer is readily peeled off. (5) The sample is dip-coated in a pH-responsive polymer

In this paper, we present a passive freestanding hybrid packaging technology for targeted exposure of ingestible capsule components that can maintain the unsupported coverage of millimeter-scale openings in a way that pH-responsive polymer coatings alone cannot. This strategy utilizes a flexible pH-responsive Eudragit^®^ FL 30 D 55 layer paired with a rigid water-soluble polyethylene glycol (PEG) support layer to form a freestanding region-responsive bilayer (FRRB), which can protect and selectively expose the millimeter-scale openings in ingestible capsules. The PEG support, formed by melt processing, provides a conformable substrate for forming geometries that were previously unattainable using dip coating. The rigid PEG support also allows the FRRB to protect the underlying components without contacting them while facilitating exposure in response to pH-specific regions along the GI tract. The bilayer eliminates the need for complex space- and energy-intensive opening mechanisms^6,9^. Without the rigid freestanding layer, the opening sizes and geometries of capsules are limited by the constraints of conventional dip coating or space and energy considerations. The operation of the FRRB occurs sequentially, where the Eudragit layer is removed in the small intestine target environment via a combination of dissolution and abrasion from the intestinal wall, then the dissolution of the PEG layer in the surrounding aqueous media occurs to reveal the underlying components (Fig. 1a). The novelty of this work is the combination of pH-responsive and water-soluble materials to attain millimeter-scale freestanding structures that are more geometrically versatile than standard coating solutions alone and less demanding than mechanical systems. Thus, the FRRB demonstrates the potential of passively activated functional packaging components. The effectiveness of the bilayer in protecting against internal leakage is evaluated through incubation in simulated GI fluids. We demonstrate the application of the FRRB by creating four capsule designs and packaging a simple heat-activated actuator that deposits a dye-infused microneedle patch onto the simulated GI tissue. This technology can provide an accessible packaging solution for ingestible microsystems that both improves the versatility of pH-responsive coverings and conserves space and energy compared to other technologies.

## Results

### Bilayer characterization

The FRRB is composed of a water-soluble rigid PEG layer under a pH-responsive Eudragit FL30 D 55 layer. Once the capsule reaches the target environment, i.e., the small intestine, the Eudragit layer is removed via a combination of dissolution and abrasion from the intestinal wall. The PEG layer is then dissolved by the aqueous media present in the small intestine (Fig. 1a). The FRRB was fabricated using easily accessible techniques, starting with film transfer of PEG and followed by dip coating in Eudragit FL30 D 55, to establish a freestanding film structure that could be applied to seal millimeter-scale capsule cavities in a manner that commercially available enteric coatings alone cannot (Fig. 1c). PEG was melted onto a Kapton^®^ film and applied over the capsule opening. The film was then wrapped tightly around the capsule to significantly reduce the development of heterogeneity during film formation. The poor adhesion of solid PEG to the transfer film ensured that this film could be easily removed without disturbing the PEG layer. This was followed by dip coating in the Eudragit FL30 D 55 liquid coating formulation. To evaluate their ability to form a sufficient coating layer over capsule openings, the FRRB and standard enteric coatings, Eudragit FL30 D 55 (FL30) and L 100 55, were applied to square capsule openings ranging from 2 × 2 mm to 6 × 6 mm of 3D-printed polyethylene terephthalate glycol (PETG) capsules. FL30, a 30% w/v aqueous suspension, was applied as purchased. L 100 55 was dissolved in solvent at high concentrations (30 and 40% w/v). The capsules were dip-coated in high-viscosity enteric coating solutions, which has been shown to be an effective capsule coating method for preparing small capsule openings for sampling in the small intestine^12,14,15^. Neither the FL30 liquid dispersion nor the highly viscous 30 and 40% w/v formulations of L 100 55 formed coatings on the 2 × 2 mm square openings as they lacked the necessary viscosity to form a freestanding film before solvent evaporation. The primary structural PEG layer of the FRRB easily formed coatings on the openings, even the largest 6 × 6 mm square opening, via the melted film transfer process. This was followed by dip coating in the pH-responsive FL30 dip solution. The plasticized FL30 formulation was chosen as the pH-responsive coating layer of the FRRB to ensure that no organic solvents were included in the films and to avoid the potential cracking that is known to occur in thin layers of unplasticized formulations^12,27^.

**Fig. 2:**
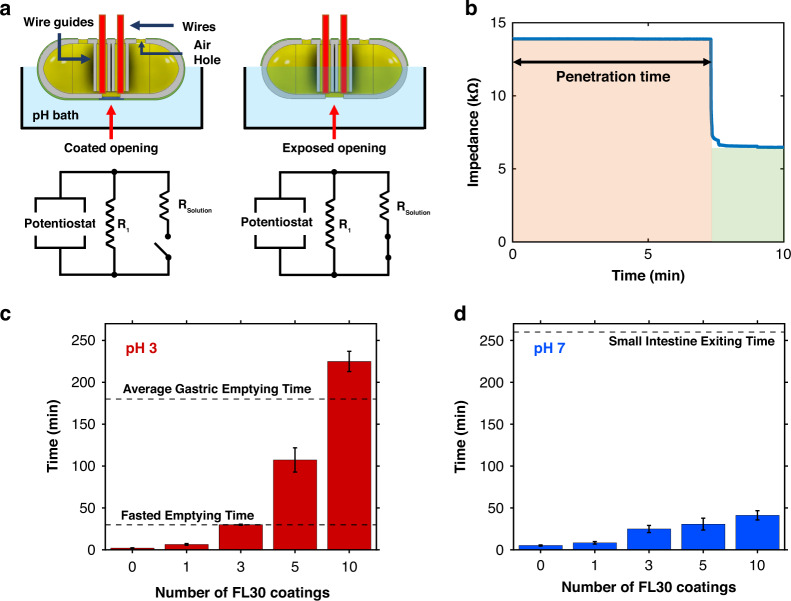
Bilayer Penetration Experiments. **a** (Top) A cross-section of coated test capsules prior to and after penetration. (Bottom) Circuit schematic of the test setup. **b** Example of the raw impedance data collected during the bath penetration experiments (1x coated sample), showing the sudden drop in impedance, indicating penetration time. **c** Bar graph of acetate penetration of the bilayer with multiple pH-dependent coating layers (0, 1, 3, 5, 10). The *x*-axis represents the number of Eudragit FL 30 D-55 layers used in the coating of the sample. The error bars represent the standard deviations. The dotted line at 180 min shows the average gastric emptying time expected with a normal fed system^46^, and the dotted line at 30 min marks the average gastric emptying time with a fasted system^47^. **d** Bar graph of the penetration time in neutral pH. The dotted line at 260 min denotes the average small intestinal emptying time (*n* = 3)

The ability of the FRRB to protect the internal capsule components against GI environmental fluid exchange was evaluated by incubating coated capsules in pH baths simulating GI transit and measuring the time to bilayer penetration. Capsules were evaluated in a simulated stomach bath at pH 3 and a simulated intestine bath at pH 7. Wires were inserted into the test capsules and positioned against the internal surface adjacent to the capsule opening (Fig. 2a). These wires were connected in parallel to a potentiostat that measured circuit impedance throughout the incubation period. The penetration time of the FRRB was determined as the time at which a sharp decline in impedance was observed, resulting from the electrical connection across the wires formed by the pH bath fluid (Fig. 2b). Capsules were dip-coated with multiple layers of FL30 (0, 1, 3, 5, and 10) to modulate the penetration time, as previously shown with similar enteric coatings^14,15,34^. GI transport can differ based on the state of the GI system prior to ingestion. Ingestible capsules are generally administered to patients in a fasted state, in which there is no food present prior to administration^46,47^. In simulated gastric fluid, capsules dip-coated in FL30 at least three times demonstrated a sufficient protection time, 29.9 ± 1.3 min, to allow for transport to the small intestine within a fasted system (Fig. 2c)^46^. For normal fed systems, gastric transport time is approximately 2 hours^46^. To achieve sufficient protection for the average fed system, the capsule would have to be coated nearly ten times, for which the penetration time was shown to be 225 ± 24 min. In the simulated intestinal environment, the FRRB was penetrated long before the estimated intestinal emptying time of 260 min, as the ten FL30 layer coating was penetrated at 41.3 ± 11 min (Fig. 2d). The mean FRRB penetration time and standard deviation for all evaluated numbers of FL30 layers are summarized in Figs. S3, S4.

**Fig. 3:**
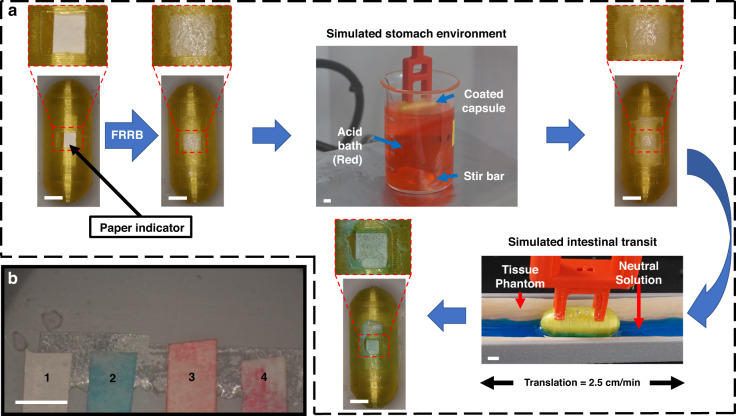
Simulated Intestinal Transit Experiments. **a** A capsule with a white paper indicator packaged inside was coated with the FRRB (5x FL30). Then, the capsule was incubated in an acid bath (pH 3). It was then translated across the tissue phantom in neutral solution (pH 7). **b** Paper strips protected/released by the capsule after four conditions: (1) Blank/no incubation; (2) 30 min of acid incubation and exposure in neutral solution on the GI simulator. (3) Negative control/acid solution. (4) Both acid and neutral solutions. (All scale bars: 5 mm)

The region-targeted exposure of the capsule contents via the FRRB was demonstrated using solution color indicators to reflect whether exposure occurred in the acid bath or neutral intestinal phantom. To do this, a paper strip was packaged inside a 5x-coated capsule, and the capsule was subjected to simulated transit through the GIT using reported ingestible capsule transit conditions^9,47^. First, the capsule was incubated for 30 min in a stirred acidic bath, simulating gastric passage in a fasted system (Fig. 3a). The capsule was then translated across simulated GI tissue in neutral solution to simulate intestinal transit. The acidic solution was dyed red, and the neutral solution was dyed blue to indicate the region of exposure. Visual inspection of the capsule showed that the bilayer remained intact and unpenetrated after acidic incubation; however, it was exposed after 5 min of transit, as indicated by the paper strip being stained blue after translation (Fig. 3b).

### Capsule geometry

**Fig. 4:**
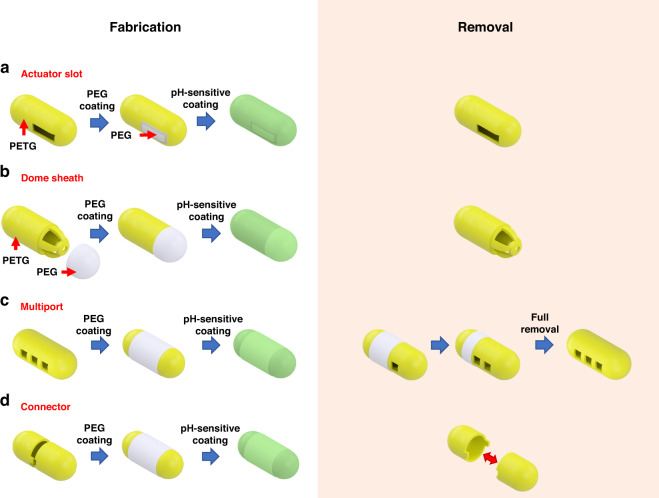
CAD designs of select functional capsule geometries possible with the FRRB. **a** Actuator slot design used to demonstrate the release of the capsule actuator for GI wall access. **b** Dome-shaped sheath that can be molded to cap the end of a capsule, leaving room for large-area openings. **c** Multiport capsule with three layers of ports that are sealed by the FRRB and exposed at different times. **d** FRRB used to connect two halves of a capsule, which are then separated due to coating removal

The FRRB can be customized to cover any portion of an ingestible capsule and to form packaging that passively serves a role in capsule operation. This could include operations such as the deposition of payloads and exposure of sensors with large surface areas. Coating is facilitated by the formation of the PEG layer into 3D shapes by molding and casting. To demonstrate the geometric versatility of the FRRB, the coating technology was applied in several functional packaging designs. The actuator slot design (Fig. 4a) was realized using the previously described FRRB fabrication process. The capsule was dip-coated in FL30 three times, as previously reported data has indicated that 3 layers would be the minimum number of layers necessary to withstand 30 min of gastric transit. Such a capsule configuration could be useful in facilitating selective exposure for actuators that require direct contact with the intestinal wall over large surface areas. The FRRB can also be applied to capsules designed with functional components that are configured to operate at the ends of the capsule. To demonstrate this, a capsule with a dome-shaped sheath geometry was fabricated and characterized (Fig. 4b). This dome-shaped sheath was molded from PEG using a PDMS negative mold and fitted over a printed capsule, which was then dip-coated in FL30 three times. Moreover, a multiport capsule was printed and PEG-coated as described in the previous section. It was then dip coated utilizing a wrapping film masking technique to individually coat each opening with a different number of FL30 layers (Fig. 4c). The ports were coated with 1, 2, and 3 layers of FL30 to induce the sequential exposure of the three cavities. This design could be used to release payloads at different positions in the GI tract with a single capsule. For the connector configuration (Fig. 4d), two halves of a capsule were connected via the FRRB coating utilizing the standard coating procedure previously described (3 FL30 layers). The capsule was configured such that exposure to the pH of the small intestine and abrasive forces in the target environment would cause the halves to separate. The connector design could be used in depositing capsule content for applications such as long-term residency.

### Evaluation of functional packaging geometries with a simulator

**Fig. 5:**
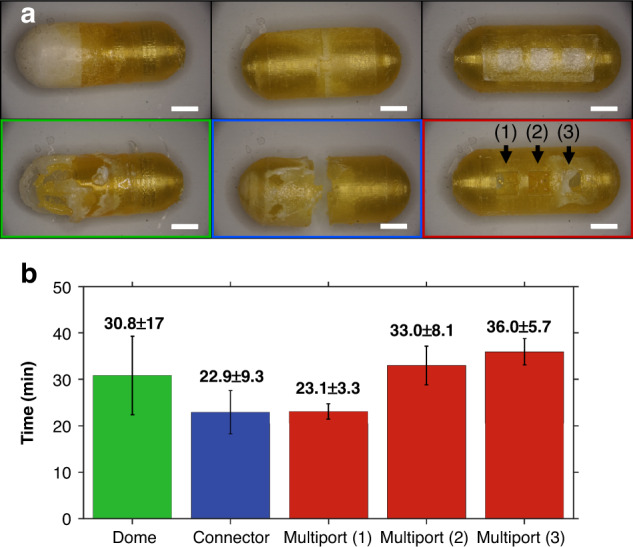
FRRB Functional Capsule Geometry Removal. **a** Images of capsules before (top) and after (bottom) translation across a tissue phantom. **b** Removal times of each geometric design, including the total time to exposure of each port of the multiport capsule from the start of translation. (*n* = 3; all scale bars: 5 mm)

To validate the ability of the capsule configurations to expose the capsule cavity in the target environment, the dome, connector, and multiport capsules were evaluated in a GI translational simulator translated across a small intestine tissue phantom to assess the coating removal time. Each of the functional packaging configurations demonstrated FRRB removal in under 45 min, suggesting their potential for targeted exposure within transit through the small intestine (Fig. 5a, b). The openings on the multiport capsule were exposed in order from the fewest FL30 coatings to the greatest FL30 coatings, as expected.

### Capsule actuator protection and selective exposure

**Fig. 6:**
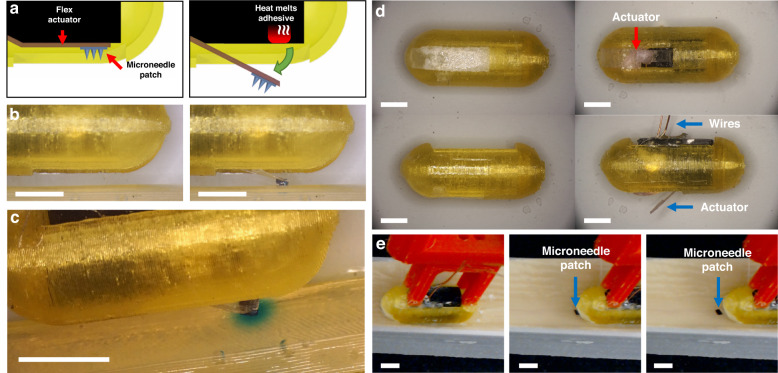
Protection and Selective Exposure of Capsule Actuator. **a** Schematic of the internal flex actuator (left) before and (right) after deployment via thermal actuation. **b** Uncoated actuator packaged capsule before (left) and after (right) firing of the actuator. **c** Image of the microneedles fired into agar showing dye diffusion from the microneedle patch. **d** Preloaded capsule coated with five FRRB layers before (left) and after (right) translation and deployment of the GI simulator. **e** Images of the capsule during translation across the small intestinal phantom showing the microneedle patch left behind as the capsule translates. (all scale bars: 6 mm)

To demonstrate the use of the FRRB to protect an active ingestible capsule component, a simple thermomechanical actuator (Fig. 6a–c) for drug delivery was integrated into a test capsule and packaged with an FRRB with the actuator slot geometric design (Fig. 4a). The actuator drug delivery system was composed of a microneedle patch adhered to a flex beam cantilever via PEG. The cantilever was fixed to a heater via a low-melting-point adhesive. The test capsule was subjected to the aforementioned fasted GI transit conditions and inspected for signs of coating removal. Once the FRRB was removed in the target region, the actuator was deployed onto the simulated tissue. The coated capsule showed no signs of penetration or internal liquid retention after the 30 min incubation period in the acid bath. After 20 min of translation across simulated intestinal tissue in neutral pH solution, the FRRB coating was fully removed, and the actuator was released (Fig. 6d). Once deployed, the microneedle patch was deposited on the phantom as the thin PEG layer adhering it to the actuator was dissolved in the fluid (Fig. 6e). Additionally, no excess blue dye was observed to be released from the capsule into the liquid prior to deployment of the actuator, affirming that the FRRB adequately protected the internal components throughout translation.

## Discussion

In this work, we demonstrated the characterization and validation of a versatile and accessible bilayer packaging technology for the protection and targeted exposure of ingestible capsule functional components. The strategy required no active stimulation and was readily facilitated via facile fabrication techniques, such as dip coating, molding, and film transfer. Neither of the materials of the FRRB have been reported to have significant electromagnetic shielding properties and thus can allow wireless communication for potential real-time in situ sensor feedback or data transmission. The vast molding possibilities of the rigid PEG layer allowed the FRRB to be formed into a myriad of shapes that were used to create the functional packaging components of an ingestible capsule. This was demonstrated by fabricating and evaluating four different capsule designs coated with the FRRB. All four designs, on average, were found to expose the internal capsule components in under 40 min of transit at neutral pH, implying the potential for release long before the small intestine exiting time (260 min)^47^. This is comparable with the total dissolution time of other nonfreestanding pH-responsive films used for small intestinal targeting^12,48^. The performance of the FRRB outside of normal physiological conditions has yet to be explored; however, we expect that a reduction in physiological pH or contact force would result in prolonged removal. A reduction in pH would decrease pH-responsive film expansion, while a reduction in contact force would decrease shearing. In the inverse case, we expect expedited removal. The characterization of the FRRB confirmed that increasing the number of film coatings increases the time to internal leakage, as was previously demonstrated by our group with other Eudragit formulations^14,15^. Thus, the FRRB could be tuned to target specific subregions of the intestines by modulating the dip-coating procedure or utilizing an alternate pH-responsive polymer formulation, such as Eudragit S 100, for large intestinal targeting^19^.

The performance of the FRRB in the GI environment depends on the dynamic array of conditions, such as temperature, pH, and mechanical abrasions, that a capsule is subjected to that contribute to film leakage and removal. The experimental methods employed in this work were designed to simulate the GI conditions of a fasted state, the state in which endoscopic capsules are administered, on the benchtop. The journey through the GI tract from the point of entry of the esophagus to the point of exit out of the large intestine involves changes in pH and mechanical perturbations; however, the body temperature is well maintained at 37 °C throughout^10^. On average, the time for transit through the esophagus is less than a minute at a neutral pH, which would have negligible effects on the FRRB given the results of the bilayer penetration and translation experiments. The stomach environment poses little challenge to the removal of the FRRB given its acidic pH and minimal interfacial abrasion due to its geometry and large volume relative to that of standard ingestible capsules^49^. This was reflected in the results of the bilayer penetration experiments as well as in the literature using related pH-responsive films with similar experimental means of validation^12,14,15,48^. FRRB removal begins in the small intestinal environment, where it is exposed to a neutral pH for the duration of transit and high contact forces (0.9–2.9 N/cm)^50^. By applying the mean contact force (1.9 N/cm) and translating at the mean translation speed expected in the small intestine (1.4 cm/min)^47^ in a neutral pH environment, a more complete simulation of relevant factors for removal was achieved when compared to those achieved by stirred solutions alone. In the future, in vivo evaluation of the FRRB will be used to further establish its efficacy.

At lower pH-responsive coating layer thicknesses (0–3), FRRB penetration at both acidic and neutral pH values was relatively similar, although the FL30 films were still attached after penetration in the acid bath. This may imply that the mode of transport across the coating at these ranges is more dominated by FL30 film porosity than dissolution. The penetration time quickly diverged in the 5–10 coating layer range. The bilayer penetration time may be increased by decreasing the dip speed. The dip speed used to prepare all samples (5.5 mm/sec) produced films under the viscous drag regime of dip coating, leading to impregnated pores in the film^39^. These pores could lead to the premature exposure of the PEG layer, ultimately accelerating the removal time. Decreasing the dip speed below 1 mm/sec would produce films within the intermediate or capillary regime of dip coating, reducing the number of impregnating pore structures^38,39,44^.

The FRRB penetration time did not always coincide with full exposure of the capsule cavity, as partial gaps in the FL30 layer provided access for the bath solution to dissolve the underlying PEG. This may have been due to excess plasticizer in the FL30 film delaying dissolution. However, removal in the small intestine is facilitated not only by the dissolution of the pH-responsive polymer but also by the abrasive interfacial forces between the film and GI lumen^50^. Translation on the GI simulator better approximates the removal in the target region as the polymer films expand and become more susceptible to mechanical removal in addition to dissolution. With these mechanisms of removal in mind, the FRRB design could be improved to release earlier during small intestinal transit by adding additional materials, such as mucoadhesives, to improve adhesion to GI walls. Increased adhesion would increase the effect of frictional force on the pH-responsive layer, facilitating earlier removal. Chitosan is a mucoadhesive biopolymer that has been used in pH-responsive drug delivery systems and has been shown not to affect the pH-responsiveness of underlying layers^22,24,25^.

As was shown, the FRRB can protect large areas of a capsule, establishing a gap between itself and the internal capsule components. The ability to protect large surface areas without making direct contact with the underlying components could provide a simple packaging solution for sensors that have been functionalized with fragile surface modifications, such as antibody or aptamer-based sensors. These sensors can be subject to damage or removal via exposure to harsh environments or blocked from actively binding to target molecules due to residue from unremoved plasticizers typically used with enteric coatings to modulate mechanical flexibility. Additionally, the FRRB can be used in concert with triggered site-specific drug delivery mechanisms, as was demonstrated with a thermomechanical actuator. Other methods that utilize enteric coatings for drug release rely on passive diffusion through the coating as it dissolves, which has been shown to disperse drugs over a period of approximately 20 min^48^. The modulation of release times by differentially coating various capsule openings can be tailored toward the development of controlled multirelease drug delivery capsules and resident devices. Overall, the FRRB provides a versatile packaging solution that can be readily applied in any ingestible capsule platform.

## Materials and methods

### Design and fabrication of 3D-printed PETG capsule templates

Test capsules (∅ = 13 mm, *L* = 32 mm) were designed in Autodesk® Inventor™ and 3D-printed through fused filament fabrication (FFF) with polyethylene terephthalate glycol (PETG) using a Prusa i3 MK3S + (Prusa Research, Czechia). A square opening (4 mm × 4 mm) was created along the capsule face to evaluate the dissolution of the FBBR. Four additional 2 mm diameter ports were positioned on opposite ends of the test capsule to allow entry of the two sensing wires used to indicate liquid intrusion, as well as to relieve air pressure and mitigate the formation of air bubbles that might prevent liquid penetration into the capsule opening even after layer removal. The capsules were printed with 1 mm thick internal cylindrical guide structures (inner ∅ = 2.5 mm) to secure the sensing wires near the capsule opening, as shown in Fig. 2.

### Coating procedure

Figure 1c depicts the coating process for each capsule in which approximately 0.17 g of PEG (polyethylene glycol 4000, MilliporeSigma, Burlington, MA, USA) was melted on a 1 mil polyimide film (Kapton®, DuPont, Wilmington, DE, USA) and then transferred to the capsule surface to cover the capsule opening. The PEG was allowed to solidify, and then the polyimide film was removed. Samples were dip coated in Eudragit^®^ FL 30 D-55 (generously donated from Evonik, Essen, Germany), a 30% w/v dispersion of pH-sensitive polymer and plasticizer in water, at a constant entrance and withdrawal speed of 5.5 mm/sec with an immersion time of 1 sec via a custom fabricated dipping apparatus and then allowed to dry at room temperature for 30 min. The dipping process was repeated after drying to obtain multiple layers (3, 5, 10). Wrapping films (Parafilm^TM^, Bemis^TM^, Sheboygan, WI) were used to mask portions of the capsules during the dipping step of the coating procedure and removed once the enteric coating films were completely dry.

### Determining pH-responsive polymer limits

A 30% w/v standard enteric coating solution was created by dissolving 15 g of Eudragit L 100 55 powder in 50 mL methanol and stirring at 300 rpm at a temperature of 60 °C until the solute was no longer visible. Capsules were printed with square openings with dimensions ranging from 2 × 2 mm to 6 × 6 mm, increasing in increments of 1 mm. These capsules were dip-coated in Eudragit FL30 D-55, and the FRRB was then assessed via observation to determine the coating success. Capsules were sectioned and inspected to determine whether internal leakage occurred.

### pH bath coating penetration and removal tests

The coating penetration time was evaluated by incubating coated capsule baths of 0.1 M acetate (pH 3) and Dulbecco’s phosphate buffer saline (DPBS, pH 7) at physiological temperature (37 °C). The baths were stirred via a magnetic bar at 250 rpm, and the temperature was maintained by an ETS Model 5506 Environmental Chamber from Electro-Tech Systems Inc. (Perkasie, PA, USA). Wires were inserted into the capsule through the guides, resting just outside of the opening. These wires were connected in parallel to a 20k or 100k resistor, which was connected to a VSP-300 potentiostat from Bio-Logic Science Instruments (Seyssinet-Pariset, France) (Fig. 2a). The impedance of the system at frequencies of 10 or 10 kHz was obtained via EC-Lab software from Bio-Logic Science Instruments (Seyssinet-Pariset, France) during the bath. The experiments were terminated after a large drop in the impedance was observed, indicating the liquid penetration of the capsule and connection of the two wires.

To evaluate the effect of the abrasive interactions between the GI wall and the capsule that facilitate coating removal, we utilized a custom-fabricated GI translation simulator that translated the capsule across a silicone small intestine phantom (SurgiReal, Inc., Loveland, CO, USA) (Fig. S1) in 7 mL of DPBS. The capsules were translated at an average peristaltic speed of 1.4 cm/min^47^ with 1.9 N/cm force^50^ (Fig. S2) applied downward via the sample holder.

### Capsule actuator protection and selective exposure

The thermomechanical actuator was composed of a flexible plastic cantilever beam (3 M, Saint Paul, MN, USA) and a Joule heating element. One end of the beam was fixed, and the other flexed to attach to the heating element with ethylene-vinyl acetate (EVA) adhesive (The Gorilla Glue Company, Cincinatti, Ohio, USA). A 5x-coated capsule containing the integrated actuator was first incubated in an acid bath for 30 min and inspected for penetration. Once it was confirmed that no acid had penetrated the bilayer, the actuator capsule was transferred directly to the GI simulator, where it was translated across a tissue phantom filled with 5 mL of DPBS. The capsule holder component of the GI simulator could freely translate vertically, which served to both apply 70 g (1.9 N/cm) of downward force to simulate small intestinal contact force and remove the capsule from contact with the tissue phantom for inspection (Figs. S1, S2). The capsule was visually inspected every 5 min to determine the time of bilayer removal. Once removal was confirmed, the capsule was returned to the tissue surface, and a current was applied to the resistive heater to melt the EVA adhesive, triggering the deployment of the microneedles onto the phantom.

## Supplementary information

Supplemental Material
