# Supplementary material for: Freestanding region-responsive bilayer for functional packaging of ingestible devices
Source: Microsyst Nanoeng. 2023 May 16;9:61. doi: 10.1038/s41378-023-00536-w (PMC10188515; doi:10.1038/s41378-023-00536-w)
Supplement: Supplementary file 1 — Supplemental Material [file 41378_2023_536_MOESM1_ESM.docx]

FREESTANDING REGION RESPONSIVE BILAYER FOR FUNCTIONAL PACKAGING OF INGESTIBLE DEVICES

*Michael A. Straker^1,4,5^, Joshua A. Levy^2,4,5^, Justin M. Stine^3-5^, Vivian Borbash^3^, Luke A. Beardslee^4^, and Reza Ghodssi^3-5*^*

^1^Fischell Department of Bioengineering, ^2^Department of Material Science and Engineering,

^3^Department of Electrical and Computer Engineering, ^4^Institute for Systems Research,

^5^Fischell Institute for Biomedical Devices

University of Maryland, College Park, MD 20742 USA

Supplementary Figures


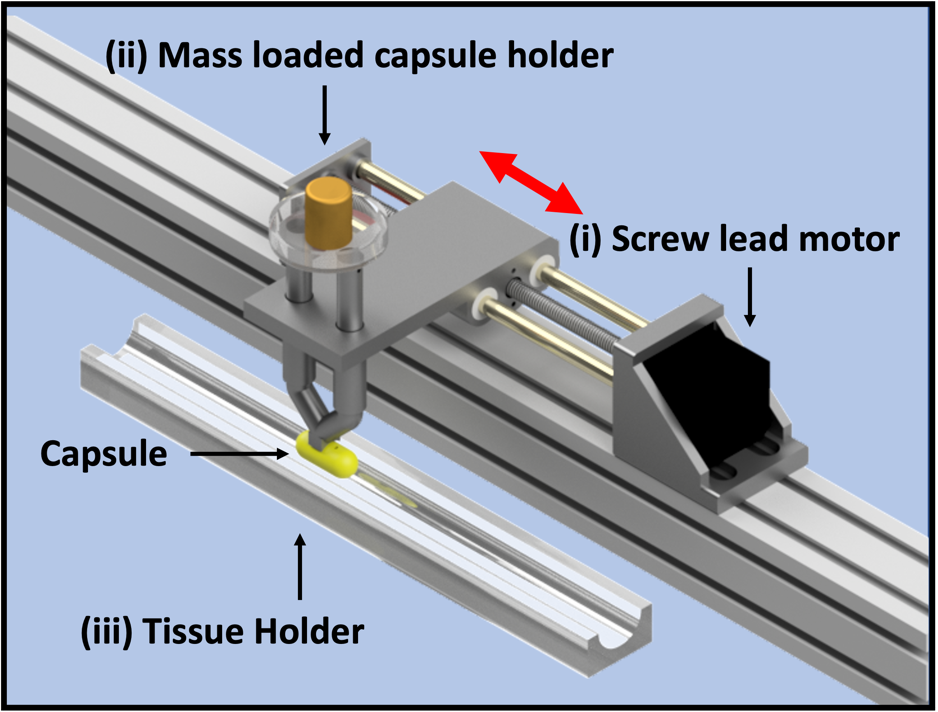


**Figure S1. GI Simulator setup.** CAD rendering of the ex-vivo intestinal simulator system. Translation is facilitated by the screw lead motor which moves the rail-mounted platform attached to the capsule holder. A dish holding a 50g mass is attached to the top of two-pronged mass loaded capsule holder while a coated test capsule is fastened to the bottom. This applies a total of ~70g of mass from the top of the capsule to the tissue phantom placed in the phantom tissue holder to approximate circumferential peristaltic forces in the GI tract.

**Figure S2. Mean Expected Contact Force Calculation.**

F_CF_ = Contact force per length (1.90 N/cm) ^49, 50^

[ℓ](https://en.wiktionary.org/wiki/%E2%84%93)_[C](https://en.wiktionary.org/wiki/%E2%84%93)_ [= Length of capsule cylinder (19 mm)](https://en.wiktionary.org/wiki/%E2%84%93)

D = Diameter of capsule (13 mm)

K = % Expected contact area (19 % or 0.19)

g = acceleration due to gravity (9.81 m/s^2^)

$$F=\left( Contact force per length \right) x \left( Length of capsule cylinder \right) x \left( Expected contact area \right)$$

$$F=\left( F_{CF} \right)\left( \mathcal{l}_{c} \right)\left( \pi D\mathcal{l}_{c}K \right)$$

$$m=\frac{F}{g}$$

Given parameters above, we can solve for the expected contact force (F), which is 0.688 N and use this result to solve the second equation. This results in an equivalent mass (m) of ~70g.


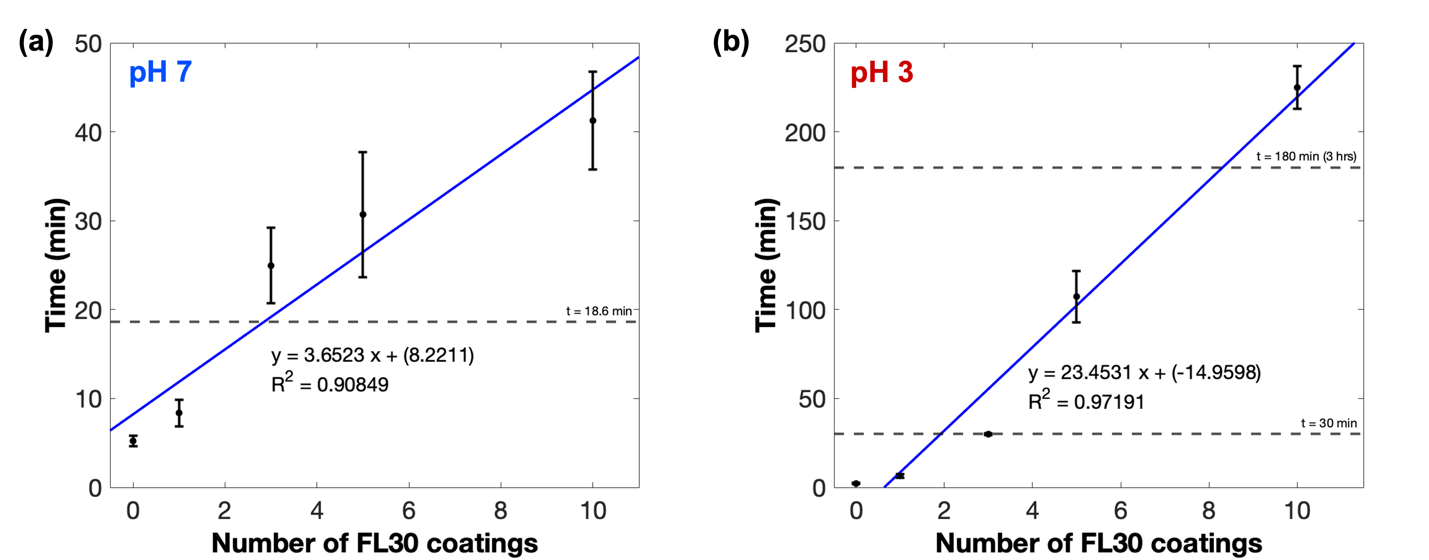


**Figure S3. Linear fit of bilayer penetration time. (a)** The dotted line represents the time of exiting the duodenum (pH 7). **(b)** The dotted line at t = 30 min represents gastric emptying in a fasted system while the dotted line at t = 180 min represents the maximum estimated gastric emptying time (pH 3).


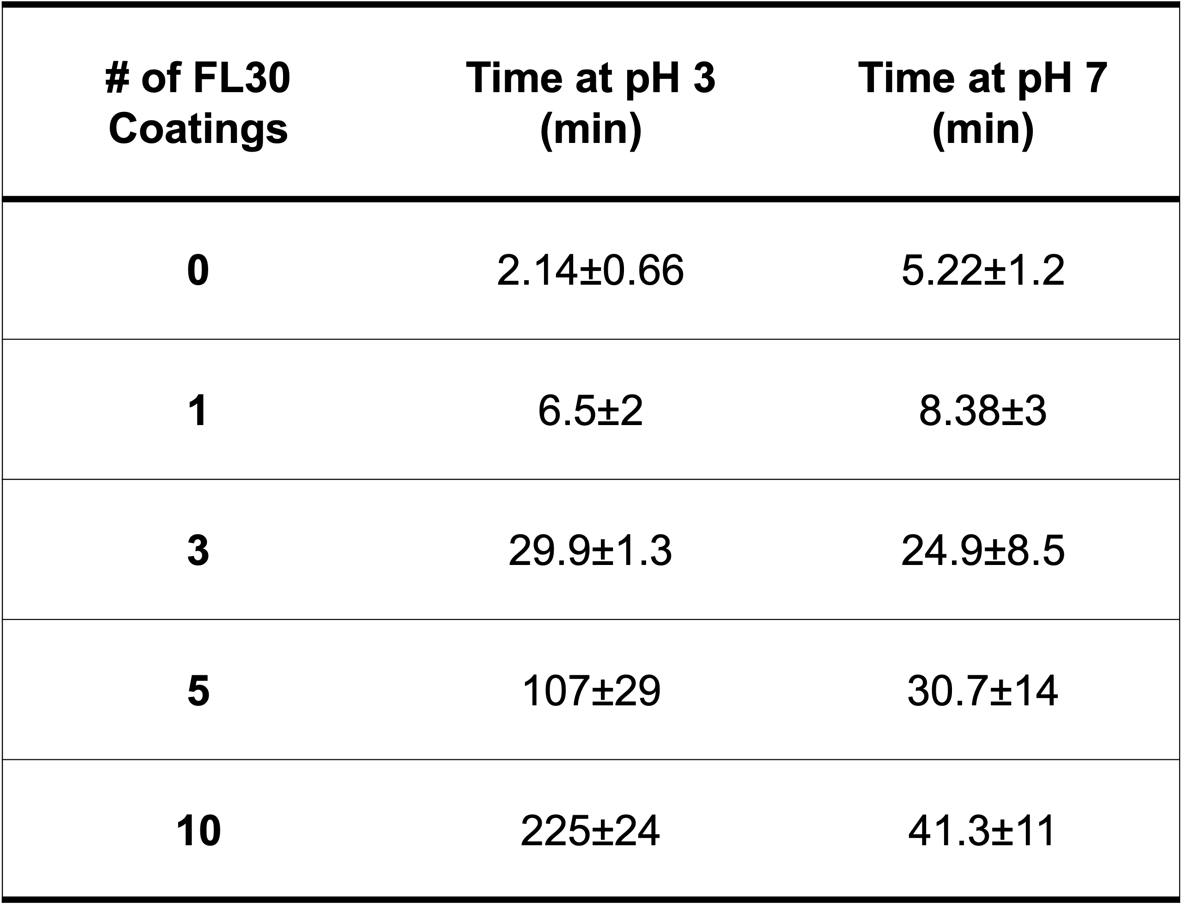


**Figure S4. Table 1:** Mean and standard deviation Freestanding Region-Responsive Bilayer (FRRB) penetration time.
